# Genetic Analysis Workshop 19: methods and strategies for analyzing human sequence and gene expression data in extended families and unrelated individuals

**DOI:** 10.1186/s12919-016-0007-z

**Published:** 2016-10-18

**Authors:** Corinne D. Engelman, Celia M. T. Greenwood, Julia N. Bailey, Rita M. Cantor, Jack W. Kent, Inke R. König, Justo Lorenzo Bermejo, Phillip E. Melton, Stephanie A. Santorico, Arne Schillert, Ellen M. Wijsman, Jean W. MacCluer, Laura Almasy

**Affiliations:** 1 Department of Population Health Sciences, School of Medicine and Public Health, University of Wisconsin, 610 Walnut Street, 707 WARF, Madison, WI 53726 USA; 2 Lady Davis Institute for Medical Research, Jewish General Hospital, 3755 Côte Ste. Catherine, Montreal, QC H3T 1E2 Canada; 3 Department of Epidemiology, University of California Los Angeles Fielding School of Public Health, Box 951772, Los Angeles, CA 90095 USA; 4 Department of Human Genetics, David Geffen School of Medicine at UCLA, 695 Charles E. Young Dr, South, Los Angeles, CA 90024-7088 USA; 5 Department of Genetics, Texas Biomedical Research Institute, PO Box 760549, San Antonio, TX 78245-0549 USA; 6 Institut für Medizinische Biometrie und Statistik, Universität zu Lübeck, Universitätsklinikum Schleswig-Holstein, Campus Lübeck, Lübeck, Germany; 7 Statistical Genetics Group, Institute of Medical Biometry and Informatics, University of Heidelberg, Im Neuenheimer Feld 305, 69120 Heidelberg, Germany; 8 Centre for Genetic Origins of Health and Disease, University of Western Australia, Perth, WA Australia; 9 Department of Mathematical & Statistical Sciences, University of Colorado-Denver, PO Box 173364, Denver, CO 80204 USA; 10 Institut für Medizinische Biometrie und Statistik, Universität zu Lübeck, Ratzeburger Allee 160, 23562 Lübeck, Germany; 11 Department of Medicine, Department of Biostatistics, Division of Medical Genetics, University of Washington, Seattle, WA 98195 USA; 12 South Texas Diabetes and Obesity Institute, University of Texas Rio Grande Valley, San Antonio, TX 78229 USA

## Abstract

Genetic Analysis Workshop 19 provided a platform for developing and evaluating statistical methods to analyze whole-genome sequence and gene expression data from a pedigree-based sample, as well as whole-exome sequence data from a large cohort of unrelated individuals. In this article we present an overview of the data sets, the GAW experience, and summaries of the contributions arranged into nine methodological themes.

## Introduction

This supplement to *BMC Proceedings* contains the proceedings of the Genetic Analysis Workshop 19 (GAW19), which was held August 24–27, 2014 in Vienna, Austria. The GAWs began in 1982 and are now held every two years. They provide a forum for statisticians, epidemiologists, geneticists, bioinformaticians, and other scientists interested in identifying genetic effects on complex diseases to evaluate and compare novel and existing statistical methods. Prior to each GAW, topics are chosen based on suggestions from previous attendees, an existing data set(s) is selected, and a set of simulated data is devised such that statistical questions of wide and current interest may be addressed. These data sets are made available to any researcher who requests them. The same data sets are provided to all researchers, thus facilitating the discussion and comparison of methods. After the GAW organizers release the data sets, researchers analyze the data and prepare a manuscript to submit to the workshop. Participation in the workshop is open to anyone who submits a manuscript, provides data, or participants in the workshop organization. More information about the GAWs, including details on upcoming meetings, can be found at http://www.gaworkshop.org.

## Genetic Analysis Workshop 19

The family dataset provided for GAW18 was used again in GAW19 with a few small corrections. New data for GAW19 included gene expression profiles for the family data set and a relatively large data set of unrelated individuals. As in past years, a simulated phenotype data set was also provided. A brief description of the data sets follows while a more detailed description can be found in Blangero et al. [1].

A family data set was provided by the Type 2 Diabetes Genetic Exploration by Next-Generation Sequencing in Ethnic Samples [T2D-GENES] Consortium. It included data from 20 Mexican American families from San Antonio, Texas, USA, with whole genome sequence information on 464 individuals. The data set also included dense single nucleotide polymorphisms (SNPs) on 959 individuals, including the 464 sequenced subjects whose genotypes served as the input for the imputation procedure. Genotype data were provided for odd numbered autosomes only, and contained sequence data, data from a genome-wide Illumina chip containing almost 500 K SNPs, and variant dosages from the Merlin-based imputation procedure. Gene expression was measured in a subset of 647 individuals using peripheral blood mononuclear cells (PBMCs) collected at the first examination and an Illumina chip. The phenotype data were longitudinal measurements of systolic and diastolic blood pressure, sex, age, year of examination, use of antihypertensive medication and tobacco smoking.

A data set of unrelated individuals was also provided by the T2D-GENES Consortium. It included 1943 Hispanic individuals (1021 T2D cases and 922 controls) with whole-exome sequence data. For this data set, only samples and variants passing extensive quality control were provided. As with the family data set, only genotype data for odd numbered autosomes were provided. The phenotypic data included the same basic traits as the family data set, but were available only at a single time point.

A simulated data set of 200 phenotype replicates was provided for both the family and the unrelated data sets. It was based closely on the real data, with the family structure (for the family data set), sex, and age taken directly from the real data. Blood pressure, medication use, and tobacco smoking were generated anew for each replicate, using the distributional structure found in the real data. The simulated values of systolic and diastolic blood pressure were influenced by over 1000 variants in over 200 genes. In addition, a normally-distributed trait, Q1, was simulated that was not influenced by any genetic variants, but was correlated between family members (in the family data set). The simulation model is described in detail in Blangero et al. [1].

The availability of the GAW19 data was announced by email in Spring of 2014 to roughly 3500 individuals on the GAW mailing list. A total of 121 groups requested GAW19 data and 87 manuscripts were submitted to GAW organizers prior to the workshop. Submitting authors were asked to select a topic that their research was most aligned with to facilitate discussion before and during the workshop. This resulted in 9 discussion/presentation groups: gene expression (Group 1), machine learning and data mining (Group 2), variant collapsing approaches (Group 3), family-based approaches (Group 4), filtering variants and placing informative priors (Group 5), methods for joint analysis of multiple phenotypes (Group 6), longitudinal analyses (Group 7), pathway-based analyses (Group 8), population-based association (Group 9). The GAW19 participants included 115 individuals from 16 countries: Australia, Belgium, Canada, China, Egypt, Germany, Hong Kong, Japan, the Netherlands, Poland, South Korea, Spain, Taiwan, Turkey, the United Kingdom, and the United States of American.

At GAW19, all groups were led by a person with previous GAW experience. This person encouraged and organized the discussion and presentations prior to, during and after the workshop. Discussions largely started before the workshop and continued at the workshop within group meetings. Each discussion group, directed by the group leader, was also in charge of preparing a presentation of the issues discussed in the group and the conclusions drawn. These presentations were made to all GAW attendees in plenary sessions. There were also two poster sessions where individual contributions could be presented.

After the workshop, participants were given just over two months to revise and resubmit their manuscript for external peer review by experts in the field. The group leader typically served as associate editor for the group. To avoid possible conflicts of interest, articles to which the group editor contributed were reassigned to other groups for the peer-review process. Of the 79 manuscripts submitted after the workshop, 57 were accepted for publication in this issue of *BMC Proceedings*. The papers are organized according to the group they were in, preceded by the data description by Blangero et al. [1].

The nine GAW19 group leaders each summarized the contributions to their group and reviewed the relevant literature in short manuscripts published in a supplement to *BMC Genetics*. These 9 summary papers, with their short reviews of the state of each field, will provide a useful entry point to researchers working with genomic data. A summary of these papers follows.

The summary paper on family-based approaches led by Wijsman [2] provides a brief history of family-based genetic studies and describes how and why such studies are enjoying resurgence, partially due to the enrichment of rare causal genetic variants in such samples. The specific topics addressed in the contributions varied widely, from initial study design questions, through quality control to many aspects of data analysis, and found numerous benefits associated with studies of carefully selected related individuals.

Two groups discussed issues around leveraging external information. In the group led by Bailey [3], they provide an overview of concepts and commonly used approaches for annotating variants in the genome, as well as a survey of several principles that are used for filtering or restricting analysis to only a subset of the variants. The participants found, in general, that appropriate choices of filters or priors increases power, not only due to increasing the sizes of true signals, but also due to reducing the number of tests performed or the proportion of null tests. The topic of pathway analysis was addressed in the group led by Kent [4]. Some participants in this group developed new approaches to pathway analysis, and many used the simulated data to assess performance. Many in the group experienced challenges in coping with the dimensionality of the data and, due to the imperfections of the required databases usually required for pathway analysis, the generalizability of identified pathways was a concern.

Tests of association between genetic data and phenotypes were discussed in several of the working groups. The summary paper from the population-based association group led by Bermejo [5] presents various new methods for testing association, as well as numerous strategies for coping with the large number of sequence-identified rare variants and how to decide on validation strategies. Particular attention was drawn to problems of estimation and convergence with sparse data, particularly when additional covariates are being explored. A useful table is provided that contains references for most key software and methods used by the group. In the summary paper from the rare variant tests group led by Santorico [6], an extensive review of methods is provided followed by carefully placing the 6 contributions into the resulting analytic framework. The discussion highlights the need for future extensions and generalizations of the concept of collapsing tests. Melton led a group that addressed tests of association with longitudinal phenotypes [7]. This paper describes recent publications on longitudinal data, the computational challenges, and benefits in power and understanding resulting from appropriate longitudinal analyses. Contributors to a group led by Schillert, developed and compared methods for analysis of multivariate phenotypes [8]. They frame the heterogeneous terminology and goals that are in use for analysis and interpretation of multivariate phenotypes. Although the approaches used by participants were diverse, all showed potential, both in terms of power and computational feasibility.

Cantor led the group studying methods for analysis of gene expression data [9]. The paper provides a quick yet broad overview of the ways in which gene expression data have typically been analyzed since high-throughput arrays became accessible, as well as important design and analysis issues. One of the key issues addressed by most group members was how to utilize gene expression measures taken from related individuals. Finally, a group on machine learning and data mining led by König [10] also addressed questions around data integration. Their paper is organized around key messages such as the benefits of integrating data of different types and the computational limitations. For each of the messages, a brief introduction presents some key references prior to introducing the work of the group.

Overall GAW19 generated many interesting discussions and some conclusions concerning the analysis of human sequence and gene expression data. These discussions also highlighted areas in which further methodological development is needed.
